# Vertical Stratification of Dissolved Organic Matter Linked to Distinct Microbial Communities in Subtropic Estuarine Sediments

**DOI:** 10.3389/fmicb.2021.697860

**Published:** 2021-07-20

**Authors:** Wenxiu Wang, Jianchang Tao, Ke Yu, Chen He, Jianjun Wang, Penghui Li, Hongmei Chen, Bu Xu, Quan Shi, Chuanlun Zhang

**Affiliations:** ^1^State Key Laboratory of Marine Geology, Tongji University, Shanghai, China; ^2^Department of Ocean Science and Engineering, Southern University of Science and Technology, Shenzhen, China; ^3^School of Environment and Energy, Shenzhen Graduate School, Peking University, Shenzhen, China; ^4^State Key Laboratory of Heavy Oil Processing, China University of Petroleum, Beijing, China; ^5^State Key Laboratory of Lake Science and Environment, Nanjing Institute of Geography and Limnology, Chinese Academy of Sciences, Nanjing, China; ^6^School of Marine Science, Sun Yat-sen University, Guangzhou, China; ^7^Southern Marine Science and Engineering Guangdong Laboratory, Zhuhai, China; ^8^State Key Laboratory of Marine Environmental Science, College of Ocean and Earth Sciences, Xiamen University, Xiamen, China; ^9^Shenzhen Key Laboratory of Marine Archaea Geo-Omics, Southern University of Science and Technology, Shenzhen, China; ^10^Southern Marine Science and Engineering Guangdong Laboratory, Guangzhou, China; ^11^Shanghai Sheshan National Geophysical Observatory, Shanghai, China

**Keywords:** FT-ICR MS, metagenomic, benthic archaea, recalcitrant DOM, estuarine sediment

## Abstract

Dissolved organic matter (DOM) provides carbon substrates and energy sources for sediment microbes driving benthic biogeochemical processes. The interactions between microbes and DOM are dynamic and complex and require the understanding based on fine-scale microbial community and physicochemical profiling. In this study, we characterized the porewater DOM composition in a 300-cm sediment core from the Pearl River estuary, China, and examined the interactions between DOM and archaeal and bacterial communities. DOM composition were highly stratified and associated with changing microbial communities. Compared to bacteria, the amplicon sequence variants of archaea showed significant Pearson correlations (*r* ≥ 0.65, *P* < 0.01) with DOM molecules of low H/C ratios, high C number and double bond equivalents, indicating that the distribution of archaea was closely correlated to recalcitrant DOM while bacteria were associated with relatively labile compounds. This was supported by the presence of auxiliary enzyme families essential for lignin degradation and *bcrABCD, UbiX* genes for anaerobic aromatic reduction in metagenome-assembled genomes of Bathyarchaeia. Our study demonstrates that niche differentiation between benthic bacteria and archaea may have important consequences in carbon metabolism, particularly for the transformation of recalcitrant organic carbon that may be predominant in aged marine sediments.

## Introduction

Dissolved organic matter (DOM) consists one of Earth’s largest carbon exchangeable reservoirs, having major significance in biogeochemical cycles. In anaerobic sediments, the decomposition of particulate-attached organic matter to DOM by hydrolysis and depolymerization is a step-wise process and serves as the principal energy and carbon source for benthic organisms (Burdige and Komada, 2015; Mahmoudi et al., 2017). However, mechanisms driving DOM-microbes coupling are largely unknown. DOM is highly complex in composition and consists of a mixture of organic compounds with different mass size and functionality (Burdige and Komada, 2015; Canuel and Hardison, 2016). The major composition of DOM is uncharacterized due to limitation of extraction and analytical methods. Nevertheless, advanced spectroscopic tools like Fourier transform ion cyclotron resonance mass spectrometry (FT-ICR MS) is capable of resolving thousands of DOM compounds with high mass resolution and accuracy and provides significant insights into the molecular composition of DOM (Oni et al., 2015; Schmidt et al., 2017).

The dynamics of DOM-microbe interactions could be investigated from geochemical and microbial aspects. Geochemical studies have examined vertical variation of porewater DOM in marine sediments, which showed the increase of recalcitrant carbon such as autochthonous humic-like fluorescent components, carboxyl-rich alicyclic molecules (CRAM) and aromatic compounds (Oni et al., 2015; Chen et al., 2017; Fox et al., 2018). The observed stratification of porewater DOM may attribute to selective biodegradation of labile DOM as well as diagenetic transformation of organic carbon (Chen et al., 2017; Schmidt et al., 2017). Consequently, refractory DOM constitutes the majority (>97%) of the total porewater DOM in anaerobic sediments (Burdige et al., 2016). However, refractory DOM could still be available for organisms in deep layers with regard to the consumption of O-rich molecules in sulfate-depleted conditions (Oni et al., 2015; Schmidt et al., 2017; Gan et al., 2020). Abiotic processes like adsorption/desorption of DOM compounds on redox-sensitive minerals may affect DOM composition but appear to be quite slow (Burdige, 2007; Chen and Hur, 2015). Thus, porewater DOM undergoing diverse interactions is dynamically changed in its composition and concentration, which requires further understanding.

Meanwhile, microbial studies have examined stratified distribution of microbial structures on a global scale (Parkes et al., 2014; Petro et al., 2017); for example, Gamma-, Delta- and Alphaproteobacteria account for a high fraction of the microbial community in surface sediments, while bacterial phyla including Chloroflexi and Planctomycetota, archaeal members Bathyarchaeia often dominate in subsurface sediments. Roughly 90% of the microbial activity may occur in subsurface sediments (Parkes et al., 2014), in which DOM-microbes interactions are diverse and fundamental for benthic carbon cycle (Kujawinski, 2011). Bathyarchaeia and Chloroflexi have been proposed to play a role in the degradation of recalcitrant organic carbon (Wasmund et al., 2014; Yu et al., 2018; Saw et al., 2020). Co-existence of highly unsaturated DOM molecules and Bathyarchaeia, Chloroflexi was also detected in subsurface sediments from Helgoland Mud Area (Oni et al., 2015). Moreover, a significant correlation between archaea and humic-like fluorescent DOM (FDOM) was observed in our previous work (Wang W. et al., 2020), indicating that archaea were potentially involved in sedimentary carbon transformation. While it is well known that DOM fuels the growth of microorganisms in sediments, specific interactions between microorganisms and DOM composition are poorly known.

Estuarine sediments consist a major reservoir of organic carbon sourced from terrestrial plants, autochthonous algae, macrophytes, microbial and anthropogenic activities (Burdige, 2007; Chen and Hur, 2015; Canuel and Hardison, 2016), in which about 45% of global carbon were estimated to be buried (Hedges and Keil, 1995). In this study, we used the state-of-art approaches of FT-ICR MS to characterize DOM molecular composition in a 300-cm sediment core from Pearl River Estuary, China. This technique was combined with high-throughput sequencing such as metagenomics to further examine the linkage between DOM and microbial community structure and function. We aimed to understand (1) the factors that influence vertical composition of sedimentary DOM and the DOM variations coupled with microbial community, (2) the characteristics of DOM associated with bacterial and archaeal communities in sediments, and (3) the metabolic potential of bacteria and archaea participating in sedimentary carbon degradation. Our results revealed the vertical stratification of DOM composition and the linkages between DOM and microbial composition, the latter of which was manifested by the different roles of bacterial and archaeal communities in sediment recalcitrant carbon transformation.

## Materials and Methods

### Sample Collection

In October 2017, a sediment core of 300 cm was collected from near Guishan Island (Pearl River Estuary) (22.1315 N, 113.8055 E). This core was sectioned into 5-cm intervals for 5–100 cm and into 10-cm intervals for 100–300 cm below sediment surface. Bacterial and archaeal distributional patterns were examined by characterizing their 16S rRNA gene abundances and diversities along geochemical profile. Briefly, we detected geochemical species including NO_3_^–^, Mn(II), Fe(II), SO_4_^2–^, NH_4_^+^, dissolved inorganic carbon (DIC) and its stable carbon isotope (δ^13^C_DIC_), dissolved organic carbon (DOC), total organic carbon (TOC) and its stable carbon isotope (δ^13^C_TOC_), and FDOM (Supplementary Table 1). Amplicon sequencing were conducted using the universal prokaryotic primers 515FB (GTGYCAGCMGCCGCGGTAA) and 806RB (GGACTACNVGGGTWTCTAAT) (Caporaso et al., 2012). The used primers cover almost uniformly all major bacterial and archaeal phyla, with the coverage reaching 83.5% for archaea and 83.6% for bacteria^1^. Chemical analyses of environmental variables and 16S rRNA gene amplicon sequencing have been reported in Wang W. et al. (2020). In this study, taxonomy of amplicon sequence variant (ASV) was reassigned using the Silva 138 99% Operational Taxonomic Units database footnote^1^ to update the taxonomic classification.

### Solid-Phase Extraction (SPE) of DOM

Sediment samples were stored at −80°C before porewater extraction. Porewater samples was obtained by centrifuging ∼100 g sediments with the procedure set as 7000 rpm, 10 min and 4°C, and then filtering using a 0.22-μm Millipore PES membrane (Thermo Fisher Scientific). DOM was extracted using stryrene-divibyl-polymere type PPL cartridges (Agilent Bond Elut PPL, 200 mg, 3 mL) as described in Dittmar et al. (2008). Cartridges were activated using 6 mL MeOH (HPLC grade) and washed using at least 9 mL pH 2 ultrapure water. Porewater samples were acidified to pH 2 with HCl (G.R.) and then passed through the PPL cartridges by gravity at a flow rate of about 2 mL/min. Before DOM elution, the columns were rinsed with at least 9 mL pH 2 ultrapure water to remove the salt and dried under soft ultrapure nitrogen gas. The SPE-DOM was finally eluted with 5 mL MeOH (HPLC grade) and stored at −20°C before MS analysis.

### Molecular Analysis of DOM by FT-ICR MS

Mass spectra were obtained using a 9.4 T Apex Ultra FT-ICR MS instrument (Bruker) equipped with negative ion electrospray ionization (ESI) source at the State Key Laboratory of Heavy Oil Processing, China University of Petroleum, Beijing, China. The DOM samples were diluted to a final concentration of 100 mg/L and injected into the ESI source at a rate of 250 μL/h. The international standard substance, SRFA (Suwannee River fulvic acids) was used as quality control to optimize condition of FT-ICR MS before sample analysis (He et al., 2020). The typical operating condition for negative-ion ESI analysis was set as: 3.5 kV spray shield voltage, 4.0 kV capillary column introduced voltage, and −320 V capillary column end voltage. The ions accumulated in the collision cell for 0.2 s then transferred into the ICR cell with a 1.1 ms time-of-flight (ToF). The ion transformation parameter for the quadrupole (Q1) was optimized at m/z 300. A total of 128 scans with 4 M word size were accumulated to enhance the signal-to-noise ratio and mass spectra were evaluated in the range from 200 to 800 Da.

The mass spectrometer was calibrated using sodium formate and then recalibrated with a known homologous mass series of the SRFA. Molecular formulae were assigned to masses with s/n > 4 and mass accuracy < 1 ppm using in-house software (He et al., 2020), and those with outlier values or identified as contaminations were manual removed. Relative abundances of DOM formulae were calculated by normalizing the peak intensities to the sum of FT-ICR MS peak intensities. For each assigned formula, the double bond equivalent [DBE = 1+1/2(2C-H+N+P)] (Koch and Dittmar, 2006) and modified aromaticity index [AI-mod = (1+C-1/2O-S-1/2H)/(C-1/2O-S-N-P)] (Koch and Dittmar, 2016) were calculated as a measure for the degree of unsaturation and aromaticity. For each sample, bulk molecular parameters were calculated based on magnitude-weighted average (Sleighter and Hatcher, 2007); for example, the nominal oxidation state of carbon [NOSC = 4-(4C+H-3N-2O-2S)/C] was calculated to indicate the average oxidation state (Riedel et al., 2012) and marked as NOSC_wa_. The degradation index (I_DEG_) was calculated to assess the degradation state based on intensities of ten molecular formulae (Flerus et al., 2012). In addition, the assigned molecular formulae were further classified to seven compound categories based on their unique O/C and H/C ratios according to Yuan et al. (2017). The following ranges were used: lipids (0 < O/C ≤ 0.3, 1.5 ≤ H/C ≤ 2.0), aliphatic/proteins (0.3 ≤ O/C < 0.67, 1.5 ≤ H/C ≤ 2.2), lignins/CRAM-like structures (0.1 ≤ O/C ≤ 0.67, 0.7 ≤ H/C ≤ 1.5), carbohydrates (0.67 < O/C ≤ 1.2, 1.5 ≤ H/C ≤ 2.4), unsaturated hydrocarbons (0.7 ≤ O/C ≤ 1.5, 0 < H/C ≤ 0.1), aromatic structures (0 < O/C ≤ 0.67, 0.2 ≤ H/C < 0.7), and tannins (0.67 < O/C ≤ 1.0, 0.6 ≤ H/C ≤ 1.5). The limitation of the categorization of compound groups from FT-ICR MS should be noted, as it is only based on the elemental ratios without structural information of DOM (Li et al., 2019).

### Metagenomic Sequencing

Six samples from the depths of 5, 20, 70, 130, 200, and 300 cm were selected for metagenomic sequencing. Total genome DNA was extracted as detailed previously (Wang W. et al., 2020). DNA was sheared into 350 bp using the Covaris M220 Focused-ultrasonicator (Covaris, Woburn, MA, United States), purified using MinkaGene Gel Extraction Kit (mCHIP, Guangzhou, China) and quantified using Qubit 3.0 Fluorometer (Thermo Fisher Scientific). Illumina libraries were constructed from about 100 ng DNA using NEB Next^®^ Ultra^TM^ DNA Library Prep Kit for Illumina^®^ (New England Biolabs, United States) according to the manufacturer’s instructions and index codes were added to sequences to each sample. Sequencing was performed on an Illumina Hiseq X-ten platform using the paired end 2 × 150 bp run-type mode at the Guangzhou Magigene Biotechnology (Guangzhou, China).

### Sequence Assembly, Genomic Binning, and Functional Annotation

Paired-end reads were processed using Trim galore (v0.5.0) (Krueger, 2012) with default settings. After removing adapter sequences and reads with a length shorter than 20 bp or with average quality score lower than Q20, about 77 ∼ 96 G clean bases per sample were generated. Trimmed reads were then assembled using megahit (v.1.1.3) (Li et al., 2016) with the following options: –presets meta-sensitive for individual assembly and –min-count 1 –k-list 25, 29, 39, 49, 59, 69, 79, 89, 99, 109, 119, 129, and 141 for mixed assembly which combine high-quality reads from six depths. The quality of the metagenomic assembly was evaluated using Quast (v.4.6.3) (Mikheenko et al., 2016) and the summary statistics are provided in Supplementary Table 2.

Individual assemblies and mix assembly with contigs longer than 1,000 bp were provided as input for metagenomic binning. BASALT (Binning Across a Series of AssembLies Toolkit) (Yu et al., 2021) was used to bin contigs from all seven assemblies via MetaBAT (v.2.12.1) (Kang et al., 2019), Maxbin (v.2.2.4) (Wu et al., 2016), and CONCOCT (v.1.1.0) (Alneberg et al., 2014) with different sensitivity parameters. Genomic dereplication and reassembly modules were conducted using SPAdes (v.3.13.1) (Bankevich et al., 2012) with careful mode employed to ensure higher quality and accuracy of the individual reconstructed metagenome-assembled genomes (MAGs, simplified as “genome” below). The completeness and contamination of the genomes were then estimated using CheckM (v.1.0.11) (Parks et al., 2015) and high-quality genomes (completeness ≥ 70% and contamination ≤ 5%) were retained in this study. Phylogenetic analysis of reconstructed genomes is detailedly described in Supplementary Text 1.

Putative coding DNA sequences were predicted for each genome using prodigal (v.2.6.3, -meta) (Hyatt et al., 2012). Averagely 1,660 predicated proteins were obtained per archaeal genomes (min = 745 and max = 4,220) and 2,781 per bacterial genomes (min = 721 and max = 5,790). Quantification of these proteins across six metagenomes were performed using Salmon (v.0.7.2) (Patro et al., 2017) by calculating reads per million, which is similar to the calculation of TPM (Transcripts Per Million) in metatranscriptome. Carbohydrate-active enzymes (CAZymes) were identified using HMM models from dbCAN (Huang et al., 2018) with an e-value threshold of 1e-15 and coverage of 0.35. Predicated proteins were further blastp (e-value ≤ 1e-5, bit score ≥ 50, sequence similarity ≥ 30%) against KEGG and Uniprot databases for searching matches to aromatic degradation genes.

### Statistical Analysis

The relationships between sediment depth and indices of SPE-DOM were explored with linear and exponential models using the “basicTrendline” package (Mei et al., 2018). Linear discriminant analysis effect size (LEfSe) (Segata et al., 2011) was used to obtain DOM formulae significantly distinct between the upper and deep layers. The samples between the depth of 250 and 270 cm were unsuccessfully in sequencing and thus excluded from subsequent analysis. Non-metric multidimensional scaling (NMDS) and principal coordinates analysis (PCoA) were used to examine the changes of DOM or microbial compositions based on Bray-Curtis dissimilarity (Bray and Curtis, 1957). All available environmental parameters and the first two principal coordinate axes (that is, PCo1 and PCo2) of microbial community composition were fit to ordination of NMDS of DOM composition using envfit function with Monte Carlo permutation test (permutation = 999). Canonical correlation analysis (CCorA) was performed using the first five PCoA axes (PCo!-5) of of microbial community and DOM compositions, which explained 64.7 and 82.1% variations, respectively, as described in Osterholz et al. (2016). Optimal model of distance-based redundancy analysis (dbRDA) was obtained with variables selected through forward step-wise selection based on 999 Monte Carlo permutations (Parvin et al., 2019). Influence of environmental drivers and microbial community on DOM composition were then estimated using dbRDA variance partitioning (Zhao W. et al., 2019). The above analyses were conducted using vegan packages (Oksanen et al., 2013). Moreover, Pearson’s correlation analysis was performed between microbial ASVs and DOM molecules occurring in over one-third of the samples. Network analysis was conducted based on significant positive correlations (*r* ≥ 0.65, *P* < 0.01) between microbial ASVs and DOM molecules having a relative abundance (among total DOM molecules) greater than 0.05%. Topological properties of network were calculated in Gephi (v0.9.2) (Bastian et al., 2009). Network was generated by grouping the same taxonomic or DOM groups to provide better visualization. Modules of highly interconnected nodes were identified using MCODE (v.1.6) (Bader and Hogue, 2003) Cytoscape plug-in with default parameters in Cytoscape (v.3.6.1) (Shannon et al., 2003).

### Data Availability

Source sequencing data, reconstructed genomes, and sample information are available in the NCBI under the BioProject ID PRJNA575161.

## Results

### Depth Profile of DOM Molecular Features

**FIGURE 1:**
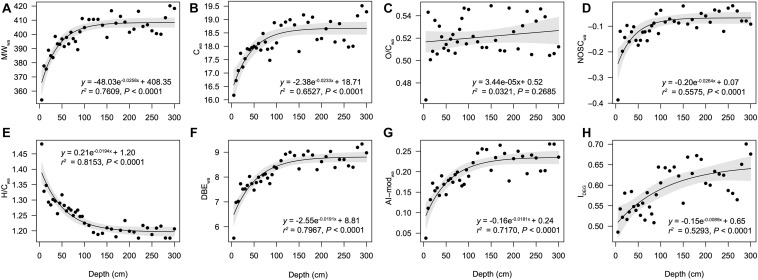
Patterns of molecular parameters of DOM along depth. Intensity weighted average values are displayed for **(A)** molecular weight (MW_wa_), **(B)** number of carbon (C_wa_), **(C)** O/C ratios (O/C_wa_), **(D)** nominal oxidation state of carbon (NOSC_wa_), **(E)** H/C ratios (H/C_wa_), **(F)** double bond equivalent (DBE_wa_), and **(G)** modified aromaticity index (AI-mod_wa_). **(H)** degradation index (I_DEG_). The relationships between sediment depth and indices of DOM molecules were modeled with linear and exponential models.

A total of 7,830 unique molecular formulae were identified via FT-ICR MS (Supplementary Table 3). The formulae number of each sample ranged from 2,840 to 5,252, and significantly increased toward deep sediments (*r* = 0.91, *P* < 0.01). Generally, the DOM compositional formulae in all but one samples were represented primarily by CHO (58.1 ± 5.5%), followed by CHON (28.2 ± 3.0%), CHOS (11.1 ± 5.9%) and CHONS (2.6 ± 1.0%), and except for the 5-cm depth, where CHOS was the most abundant compounds (36.0%) (Supplementary Figure 1A). Bulk parameters showed tremendous changes in the surface sample (Figures 1A–H) and exhibited exponential-like profiles expect for the oxygen saturation index (O/C_wa_). The molecular weight (MW_wa_) increased from 353.8 Da at surface to 420.2 Da in the deep layers and the number of carbon (C_wa_) increased from 16.2 to 19.5 downcore. The O/C_wa_ varied randomly while NOSC_wa_ showed a slight increase below 5-cm depth. The hydrogen saturation index (H/C_wa_) dropped from 1.48 at the surface to 1.19 at the 120-cm depth, and stayed relatively constant in deeper layers. The variations of these indices imply the accumulation of relatively aged and recalcitrant molecules toward the deep layers. This is further supported by the depth profiles of DBE_wa_, AI-mod_wa_, and I_DEG_, which showed the increasing depth patterns in the unsaturated, aromatic and refractory compounds. When all the molecules were classified by specific elemental compositions, lignins/CRAM-like structures dominated at all depths (Supplementary Figure 1B), averagely accounting for 69.5% (7.5%). The decreases of aliphatic/proteins (12.7 ± 5.0%), lipids (3.2 ± 3.9%), and carbohydrates (3.5 ± 1.7%) were accompanied by the increases of aromatic structures (2.1 ± 0.6%) and tannins (8.9 ± 2.0%) (Supplementary Figure 1B). Specifically, aromatic structures and tannins increased from 0.66 and 4.99% at the surface to 3.24 and 11.89% at 250-cm depth, respectively.

### Stratification of DOM Molecular Composition and Its Relationship With Microbial Community

**FIGURE 2:**
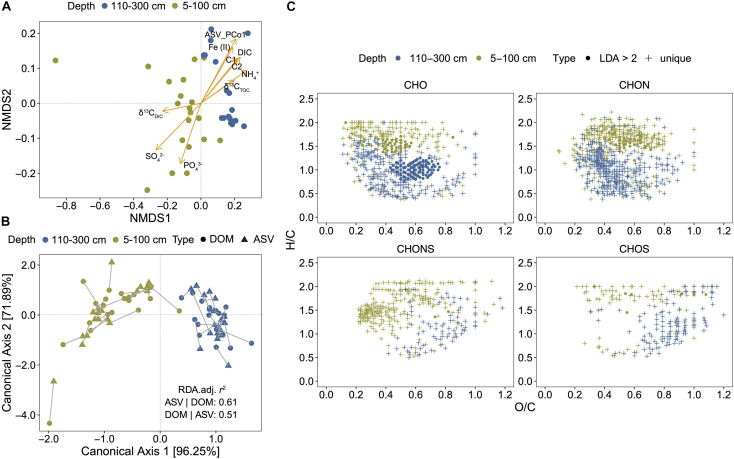
Stratification of DOM composition and its association with environmental drivers and microbial community. **(A)** Non-metric multidimensional scaling (NMDS) of DOM composition (matrix: Bray-Curtis, stress = 0.0222). ASV, amplicon sequence variant. ASV_PCo1 represents the first principal coordinate axis of microbial community. **(B)** Canonical correlation analysis (CCorA) conducted using first five principal coordinate (PCo1–5) axes of DOM composition and microbial community. The lengths of connecting lines represent the dissimilarities of two datasets. **(C)** DOM molecules unique in the upper or deep layers and different between them; different molecules were identified by Linear discriminant analysis (LDA) effect size (LEfSe) with a threshold of LDA > 2. Solid circles represent LDA > 2 and cross symbols represent compounds unique to the upper (5–100 cm) or the deeper (110–300 cm).

The NMDS based on the relative abundance of DOM molecules revealed a separation of samples with depth (5–100 cm and 110–300 cm) (Figure 2A). The explanatory variables such as SO_4_^2–^, NH_4_^+^, DIC, humic-like FDOM (C1 and C2), Fe (II), and PCo1 of microbial Bray-Curtis distances (ASV_PCo1) (*r* > 0.7, *P* < 0.01) may have significantly shaped the DOM composition (Figure 2A). dbRDA were conducted to estimate the contribution of environmental variables and microbial community in DOM composition. Three environmental factors (SO_4_^2–^, δ^13^C_DIC_, and δ^13^C_TOC_) and one microbial feature (ASV_PCo1) were selected for optimal dbRDA model (Supplementary Figure 2A). Totally 44.9% of DOM variance could be explained, in which microbial feature and environmental factors accounted for 3.15 and 15.96% (Supplementary Figure 2B), respectively; and 25.81% together. To further characterize the association between DOM and microbial compositions, CCorA was then performed using PCo1-5 axes of DOM and microbial Bray-Curtis matrices (82.1 and 64.7% of the total variance, respectively) (Figure 2B). Highly significant relationships between DOM and microbial compositions were observed by CCorA (Pillai’s trace statistic = 1.704, *P* < 0.001) with high canonical correlations found on the first two axes (0.963 for canonical axis 1, 0.719 for canonical axis 2).

Based on the depth separation of DOM composition, we divided all samples into two groups (5–100 cm and 110–300 cm). LEfSe analysis determined 210 molecules significantly contributing to the differences in DOM composition between the two groups (Figure 2C and Supplementary Table 4). Most of them belonged to CHO compounds with decreased H/C ratios and increased O/C ratios in deep layers. CHON compounds occurred only in the upper layers. In addition, there were 749 molecules unique in the upper layers, which belonged to aliphatic/proteins, lignins/CRAM-like structures or carbohydrates; and 805 molecules unique in the deep layers, which belonged to lignins/CRAM-like structures, tannins or aromatic structures (Figure 2C and Supplementary Table 4). Unique CHOS and CHONS compounds in deep layers were characterized by higher O/C ratios than CHO and CHON compounds.

### DOM Molecules Correlated With Bacterial and Archaeal Communities

**FIGURE 3:**
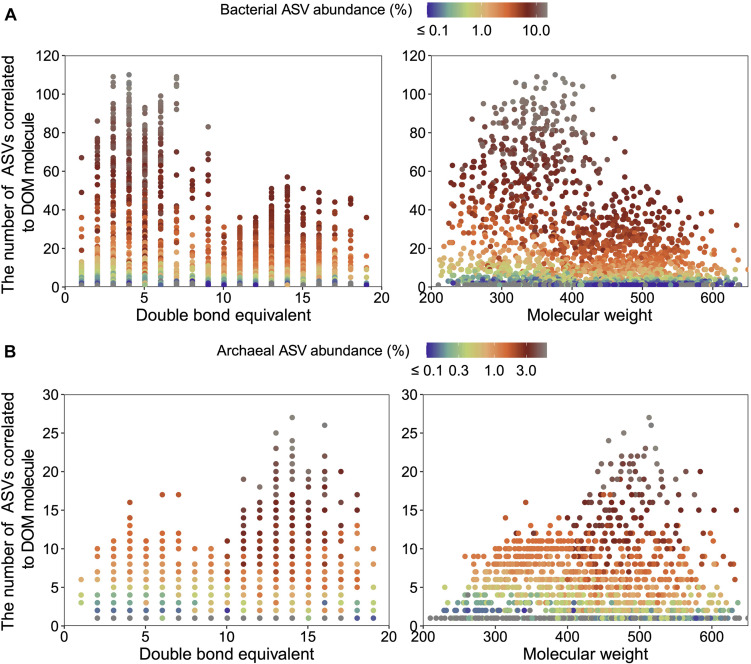
Double bond equivalent (DBE) and molecular weight (MW) of DOM molecules significantly correlated (Pearson’s correlation with *r* ≥ 0.65, *P* < 0.01) to bacterial (397 amplicon sequence variants (ASVs) in total) **(A)** and archaeal (128 ASVs in total) compositions **(B)**. Note that an ASV may be counted more than once due to its correlations with multi-molecules.

**FIGURE 4:**
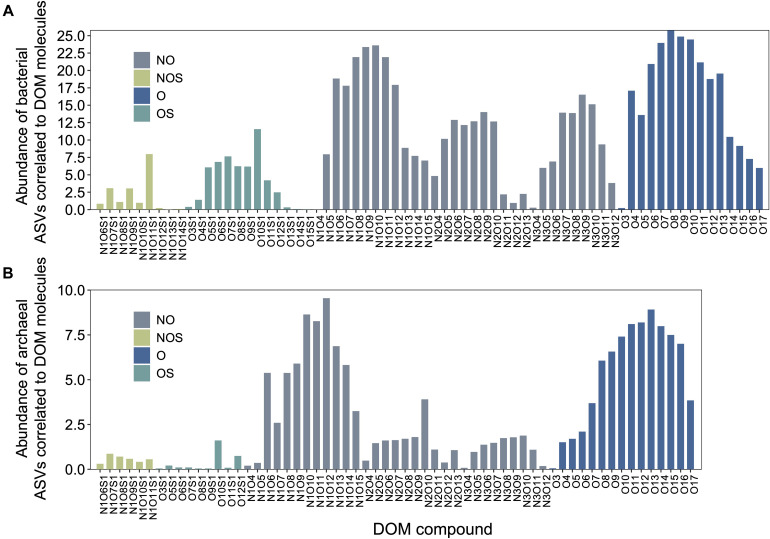
DOM compounds of molecules significantly correlated (Pearson’s correlation with *r* ≥ 0.65, *P* < 0.01) with bacterial **(A)** and archaeal communities **(B)**. ASV, amplicon sequence variant.

Microbial ASVs (1,031, accounting for 75.72% of the total abundance) and DOM molecules (4,994, accounting for 97.96% of the total abundance) occurring over one-third of the samples were selected for Pearson’s correlation analysis; molecular features (e.g., DBE and MW) of formulae correlated with bacterial and archaeal communities were then demonstrated. Pearson’s correlation analysis determined 397 bacterial and 128 archaeal ASVs significantly positively correlated with DOM molecules (*r* ≥ 0.65, *P* < 0.01). Most of bacterial ASVs were associated with molecules whose DBE was less than 10 and MW less than 450 Da, approximately (Figure 3A and Supplementary Table 5). In contrast, archaeal ASVs with increasing abundance were associated with molecules of high DBE (in the range of 11–18) and high MW (500 Da), approximately (Figure 3B and Supplementary Table 5). These results indicated distinct ecological niches of bacterial and archaeal communities though more experimental evidence are needed. Furthermore, the average values of DBE, MW, AI-mod, H/C, and O/C ratios of 2,038 bacterial-correlated and 1,593 archaeal-correlated molecules were comparable while the weighted average had substantial difference except for O/C ratios (Table 1). Moreover, DOM molecules correlated to archaea mainly belonged to the compounds of N_1_O_10_-N_1_O_13_ and O_11_-O_14_ (Figure 4B and Supplementary Table 5), while those correlated to bacteria were more distributed in the compounds of N_1_O_11_S_1_, N_1_O_5_-N_1_O_12_, O_6_-O_9_, N_2_O_X_-N_3_O_X_, and O_X_S_X_ (Figure 4A and Supplementary Table 5). Overall, bacteria-correlated DOM was more widely distributed and archaea-correlated DOM was more concentrated with the characteristics of O-enrichment.

**TABLE 1:** Average and weighted average of indices of DOM molecules significantly correlated (Pearson’s correlation with *r* ≥ 0.65, *P* < 0.01) to bacterial and archaeal communities.

**Taxonomy**		**DBE**	**MW**	**AI-mod**	**H/C**	**O/C**
Bacteria	Average	8.89	415.99	0.18	1.26	0.51
	Average SD	4.58	97.19	0.30	0.37	0.14
	Weighted average	6.83	376.30	0.07	1.42	0.52
	Weighted average SD	0.10	1.85	0.01	0.01	0.00
Archaea	Average	9.02	416.24	0.19	1.25	0.52
	Average SD	4.80	95.88	0.30	0.38	0.14
	Weighted average	10.00	429.76	0.24	1.18	0.53
	Weighted average SD	0.12	2.16	0.01	0.01	0.00

*Weighted average was calculated by weighting with number of correlated amplicon sequence variants (ASVs). DBE, double bond equivalent; MW, molecular weight;*

*AI-mod, modified aromaticity index; H/C, H/C ratios; O/C, O/C ratios.*

### Bacterial and Archaeal Groups Differentially Associated With DOM Molecules

**FIGURE 5:**
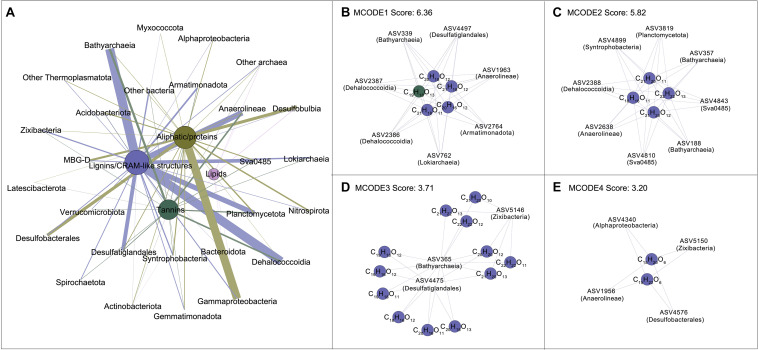
Co-occurrence patterns of microbial and DOM compositions. **(A)** Network generated by grouping with categories of original network displayed in Supplementary Figure 3 for better visualization. The nodes represent the clusters of microbial amplicon sequence variants (ASVs) or DOM molecules belonging to the same category. Node and edge sizes are proportional to the number of significant connections (Pearson’s correlation with *r* ≥ 0.65, *P* < 0.01) (degree). **(B–E)** Four sub-networks with highest node scores identified by MCODE analysis with default parameters; the nodes represent microbial ASVs or DOM molecules. The color of nodes indicates distinct microbial groups or DOM compounds; the color of edges indicates distinct targets to DOM compounds.

The DOM molecules with relative abundance over 0.05% were considered for network analysis to further explore the interconnections between DOM composition and microbial communities (Supplementary Table 6). The original network was composed of 457 nodes and 3,397 edges with an average number of neighbors of 14.85 (Supplementary Figure 3). Network was then generated by grouping categories for better visualization (Figure 5A). The lignins/CRAM-like structures consist of 120 DOM molecules showed most correlations with Bathyarchaeia, followed by Dehalococcoidia, Anaerolineae, Planctomycetota, and Desulfatiglandales, whereas the aliphatic/proteins largely associated with Gammaproteobacteria, Desulfobulbia and Desulfobacterales. Tight connections between diverse species and tannins were also found, especially in Bathyarchaeia, Anaerolinaea, and Dehalococcoidia. The remaining DOM belonging to lipids exhibited less associations with microbial species compared to others. In addition, although DOM molecules belonging to aromatic structures were not included in the network, they exhibited close correlations with archaeal groups (70 DOM molecules significantly correlated to 59 ASVs) such as Bathyarchaeia, and bacterial groups (90 DOM molecules significantly correlated to 114 ASVs) such as Dehalococcoidia.

Four significant clusters with the scores ranging between 6.36 and 3.20 (Figures 5B–E) were identified by MCODE analysis. Cluster ranks 1–3 consisted of 21 DOM molecules and 18 microbial ASVs. These molecules were enriched in the 110–300 cm depth interval, which had H/C ratios less than 1.05 and were associated with ASVs affiliated to Bacthyarchaeia, Dehalococcoidia, Desulfatiglandales and Sva0485 (Figures 5B–D). Two DOM molecules (C_15_H_20_O_6_ and C_15_H_22_O_6_) belonging to Cluster rank 4 were enriched in the 5–110 cm depth interval and correlated to microbial ASVs affiliated to Alphaproteobacteria, Desulfobacterales as well as Anaerolinaea and Zixibacteria (Figure 5E). Details of H/C and O/C ratios of different DOM molecules were showed in Supplementary Figure 4. Overall, archaeal groups including Bathyarchaeia and Lokiarchaeia were predominately associated to molecules belonging to lignins/CRAM-like structures having H/C ratios < 1.5, while MBG-D was associated to alphatic/proteins. Bacterial groups such as Gammaproteobacteria, Desulfobulbia and Desulfobacterales predominately represented correlations to molecules belonging to alphatic/proteins having H/C ratios > 1.5; however, Dehalococcoidia, Anaerolinaea, Planctomycetota, and Desulfatiglandales were exceptionally linked with lignins/CRAM-like structures and tannins.

### Potential Functions of Bacteria and Archaea in Organic Matter Degradation

A total of 51 archaeal and 166 bacterial genomes with completeness more than 70% and contamination lower than 5% were chosen for function predictions (Supplementary Table 7, Supplementary Figures 5, 6, and Supplementary Text 2). Metabolic potentials for metabolizing complex carbohydrates in archaeal and bacterial communities were investigated by searching CAZymes within each of the genomes. In total, we detected 1,219 (24 per genome) archaeal and 11,834 (72 per genome) bacterial potential CAZymes. The abundances of CAZymes in archaeal genomes generally increased along the depth profile and approached that of bacteria in the 200 and 300-cm depths. Significant difference (*P* < 0.05, Non-parametric Mann-Whitney test) existed between the bacterial and archaeal communities in upper layers (Supplementary Figure 7A and Supplementary Table 7). Glycosyltransferases (GT) and glycosyl-hydrolases (GH) catalyzing the synthesis and breakage of glycosidic bonds were the predominant CAZymes families in the archaeal and bacterial enzyme pools (Supplementary Figure 7B), which is consistent with observation in other environments (Jimenez et al., 2016; Dombrowski et al., 2018). Auxiliary activities (AA) were associated with redox enzymes essential for lignin degradation, including ligninolytic enzymes and lytic polysaccharide mono-oxygenases (Levasseur et al., 2013). Archaea encoded higher fraction (15.76%) of AA families and resulted in higher AA/GH ratios (0.86 ∼ 1.04) than bacteria (Supplementary Figures 7B,C).

**FIGURE 6:**
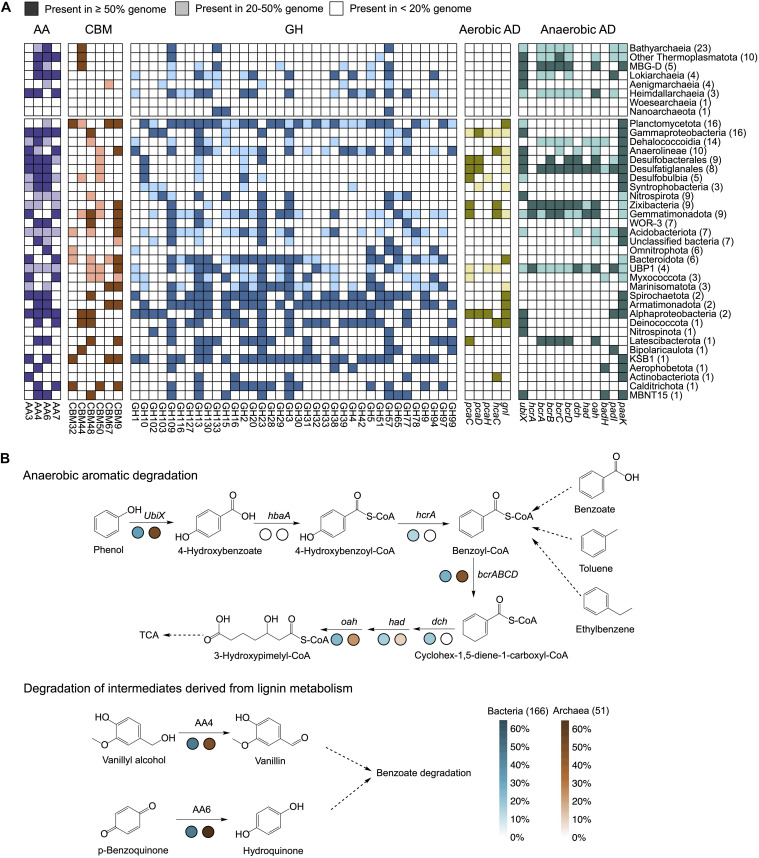
Metabolic potential involved in organic degradation of genomes. **(A)** Percentage of carbohydrate-active enzymes (CAZymes) and aromatic degradation genes encoded in genomes summarized for each cluster. Each group of enzymes were set as the same color codes strategies. Brackets: Total number of genomes in each phylogenetic cluster. AA, auxiliary activities; CBM, cellulose-binding motifs; GH, glycosyl-hydrolases; Aerobic AD, aerobic aromatic degradation; Anaerobic AD, anaerobic aromatic degradation. **(B)** Reconstructed anaerobic aromatic degradation pathway and reactions participating in lignin degradation. Percentage of genes were summarized for archaea and bacteria. Brackets: Total number of genomes in each domain.

In general, bacteria encoded a broader range of CAZymes than archaea, similar with the findings in the Guaymas Basin (Dombrowski et al., 2018). Most CAZymes identified in archaeal genomes were AA4, AA6, AA7, CBM44, GH109, and GH57 families, while AA3, CBM48, GH3, GH23, and GH77 were more common in bacteria (Figure 6A and Supplementary Table 8). These enzymes involving in carbon degradation showed obvious depth distribution accompanied with the changing of microbial community composition (Supplementary Figure 8 and Supplementary Table 9). CAZymes involving cleavages of peptidoglycan (GH13, GH23, and GH102), xyloglucan (GH16), cellobiose (AA3), glucooligosaccharide (AA7), glycogen- and chitopentaose-binding motifs (CBM48 and CBM50) were predominant in the upper layers, mainly derived from Desulfobulbia, Gammaproteobacteria, and Desulfatiglanales. CAZymes enriched in deep layers were encoded by Planctomycetota (most of GH families), Bathyarchaeia (AA6, CBM44, GH57, and GH133), and Anaerolineae (CAZymes enriched in 200-cm depth). AA6 (1,4-benzoquinone reductase) and AA4 (vanillyl-alcohol oxidase) involved in degradation of aromatic compounds in subsurface were mainly present in Bathyarchaeia and Desulfatiglanales (Supplementary Figure 9). Accordingly, cellulose-binding motifs CBM44 and CBM9 were also enriched in deep layers.

What needs to be highlighted is the diversity of aromatic metabolisms involved by both archaeal and bacterial populations from sediments. Genes involving in aerobic aromatic degradation pathways were strictly found in bacteria; for example, *pcaC, pcaD*, and *pcaH* genes were exclusively present in bacteria for the degradation of terephthalate, *hcaC* gene for the degradation of phenylpropanoate and *gnl* gene for the degradation of caprolactone. Genes that are commonly present in both archaeal and bacterial genomes include the *paaK* and *padI* genes related to the phenylacetic acid pathway and the genes encoding enzymes for hydrolytic cleavage of the ring *(dch, had*, and *oah)*. The *bcrABCD* genes targeting anaerobic aromatics degradation, however, were identified in most archaeal lineages, especially Bathyarchaeia and other Thermoplasmatota (MBG-D was excluded), suggesting the increasing importance of archaea in the utilization of ATP-dependent benzoyl-CoA reductase (BCR, class I) (Figure 6B and Supplementary Table 8). Also, the gene encoding enzyme for carboxylation (*UbiX)* was frequently detected in 50.9% of archaeal genomes and only 32.5% of the bacterial genomes (Figure 6B). Lastly, though bacteria harbored more broad aromatic degradation genes (i.e., near complete pathway for phenol metabolism in bacterial linages like Desulfatiglanales, Zixibacteria, and Gemmatimonadota), archaea especially Bathyarchaeia contained greater abundance of genes (*UbiX* and *bcrABCD*) for key processes (carboxylation and dearomatization) (Supplementary Figure 9).

## Discussion

Dissolved organic matter in sediment porewater is primarily derived from enzyme-mediated hydrolysis and depolymerization of POM and/or lysis of microbial cell components (Burdige and Komada, 2015; Liang et al., 2017). Porewater DOC concentrations generally increase with depth, which in anoxic sediments show particular exponential-like profiles (Komada et al., 2013; Chen et al., 2017; Schmidt et al., 2017). This study first characterized the exponential-like profiles of the properties of DOM molecules (MW_wa_, C_wa_, NOSC_wa_, H/C_wa_, AI-mod_wa_, DBE_wa_, and I_DEG_) in the Pearl River estuarine sediments. The stable values of these parameters below 100 cm could be explained by the balance of DOM production and consumption. It could also indicate a combination of net DOM production and decreasing bulk reactivity of DOM (Burdige and Komada, 2015), as evidenced by the accumulation of non-reactive DOM in this study (Figures 1E–H). Increasing MW as well as AI-mod, DBE, and I_DEG_ of SPE-DOM molecules down core indicated accumulation of refractory DOM as reported elsewhere (Li et al., 2019).

While carbon degradation continues with sediment depth, the spectrum of substrates becomes narrow (Jørgensen, 2000; Orsi et al., 2020) and becomes increasingly important in shaping the microbial community structure. Our study demonstrated that bacterial and archaeal communities were separated based on their correlations to different DOM compositions. Gammaproteobacteria, Desulfobacterales, and Desulfobulbia occupied their ecological niches in upper sediments and may exhibit preference for alphatic/protein groups (see also references Teeling et al., 2012; Nikrad et al., 2014; Zhao Z. et al., 2019). Dehalococcoidia and Bathyarchaeia have been thought to be important for organic matter degradation in deep sediments (Oni et al., 2015; Lazar et al., 2017; Vuillemin et al., 2020); Bathyarchaeia, in particular, were capable of lignin utilization in enrichment cultivation (Yu et al., 2018) and significantly correlated with terrestrially derived organic matter in Pearl River estuary (Wang P. et al., 2020). This is consistent with our observation that DOM molecules belonging to lignins/CRAM-like structures were correlated to Dehalococcoidia, Bathyarchaeia and other deep-sediment enrich groups like Planctomycetota and Desulfatiglanales (Figure 5 and Supplementary Figure 4). These microorganisms in deep subsurface may continuously utilize refractory DOM. Recent studies showed that CRAM organic matter such as benzoic acid and lignin were able to rapidly or slowly stimulate microbial growth (Liu S. et al., 2020). This was also supported by increased archaeal biomass especially Bathyarchaeia (Wang W. et al., 2020) and their correlations to O-rich molecules (Figure 4B).

The increasing recalcitrance of DOM represented by lignins/CRAM-like structures, tannins and aromatic structures in sediments apparently affect the strategies for substrate utilization by bacteria and archaea. Aromatic metabolism is commonly observed to be performed by sedimentary bacteria. For example, the sulfate/nitrate-reducing bacteria such as *Desulfobacula* and *Desulfobacterium* via benzoyl-CoA degradation pathway (Wöhlbrand et al., 2012; Boll et al., 2020). In recent years, aromatic degradation pathways were extended to archaeal members like MBG-D that have the phenylacetic acid pathway (Liu W.W. et al., 2020), Bathyarchaeia the protocatechuate degradation pathway (Meng et al., 2014; Zhou et al., 2018) and a few Asgard members the benzoyl-CoA degradation pathway (Farag et al., 2020). In this study, though bacteria encode a near complete pathway of aromatic degradation, archaea were possible to mediate the key processes like dearomatization and carboxylation (Figures 6A,B). BCR encoded for benzoyl-CoA pathway was frequently identified in archaeal genomes especially in Bathyarchaeia and other Thermoplasmatota (Figure 6A). The central aromatic intermediate benzoyl-CoA in benzoyl-CoA degradation pathway could derive from various monocyclic aromatic compounds including benzene, toluene, phthalates, phenylacetic acid, O-xylene, and more (Boll et al., 2020) and BCR were targeted to initiate the key reaction that dearomatizes the benzoyl-CoA to 1,5-dienoyl-CoA, which further involves a series of beta-oxidation like reactions to produces acetyl-CoA (Boll et al., 2020; Farag et al., 2020). Our study points out the prevalence of BCR in archaea, especially in Bathyarchaeia, expanding the spectrum of archaeal lineages capable of utilizing aromatic compounds as carbon or energy source. Another potential key reaction in the anaerobic degradation pathways found here could be C-H bond activation of non-substituted benzene, naphthalene or phenol by *UbiD*/*UbiX*-like genes-induced carboxylation, which were identified in benzene degrading *Ferroglobus placidus* (Holmes et al., 2011) and Fe (III)-reducing or methanogenic enrichment (Kunapuli et al., 2008; Luo et al., 2014). Archaea containing genes involving carboxylation (*UbiX*) and dearomatization (*bcrABCD*) were both of high frequency and abundance in deep layers (Figure 6B and Supplementary Figure 9), demonstrating potential importance of archaea in anaerobic aromatic degradation.

Benthic archaea may be particularly important in utilization of recalcitrant organic matter in low energy environments (see below). So far, archaea are known to degrade detrital proteins (Lloyd et al., 2013; Lazar et al., 2017), polymeric carbohydrates (Lazar et al., 2017), alkane or methylated compounds (Wang et al., 2019), aromatics (Meng et al., 2014; Farag et al., 2020; Liu W.W. et al., 2020, this study) and lignin (Yu et al., 2018). Besides, AA6 families involving in intracellular aromatic biodegradation (Levasseur et al., 2013) are identified in lignocellulolytic or cellulolytic bacterium dominated consortia (Jimenez et al., 2016; Kanokratana et al., 2018) and were in high abundance in Bathyarchaeia genomes (Supplementary Figure 9). Linolytic enzymes in the AA class generally cooperate with classical polysaccharide depolymerases targeting the plant biomass (Levasseur et al., 2013). High potential of lignin degradation may exist in archaeal communities (Yu et al., 2018), which is also supported by high AA/GH ratios and tight associations to lignins/CRAM structures in this study. It has been noted that “we are woefully unaware of DOM production (or assimilation) mechanisms in the Archaea.” (Kujawinski, 2011). A recent study has suggested that the metabolites of marine ammonia-oxidizing archaea like CRAM-like compounds may contribute to the composition of ocean DOM (Bayer et al., 2019). In this study, we highlighted the role of archaea in interacting with sedimentary non-reactive DOM considering their considerable biomass in subseafloor sediments globally (Lipp et al., 2008; Hoshino and Inagaki, 2019). Future work is needed to develop models based on cultivation combined with isotope and metatranscriptomic methods to quantify the contribution of archaea to carbon cycle in the sediments.

In summary, the molecular composition of porewater DOM showed stratification along depth in the Pearl River estuarine sediments. The recalcitrance of DOM increased below 100 cm, which was characterized by higher MW, DBE and lower H/C. Most bacteria were found to be correlated to DOM molecules with DBE < 10 and MW < 450 Da while archaea were closely correlated to molecules with the DBE between 11 and 18 and the MW of 500 Da, indicating greater correlations between archaea and the recalcitrant DOM. In particular, the archaeal group Bathyarhcaeia along with the bacterial groups Dehalococcoidia, Anaerolineae, Planctomycetota, and Desulfatiglandales were tightly correlated to the lignins/CRAM-like structures. In contrast, bacterial groups such as Gammaproteobacteria, Desulfobulbia, and Desulfobacterales were closely associated to alphatic/proteins that are more labile for degradation. These results allowed for distinction of carbon metabolizing genes from microbial genomes, with bacteria encoding a wider range of GH families of CAZymes involving glycolytic pathways while archaea a higher proportion of AA families and key genes in anaerobic aromatic degradation of predominantly recalcitrant DOM. These findings expand our knowledge about microbe-DOM relationship in sediments, particularly the role of archaea in sedimentary carbon transformation and/or perseveration.

## Data Availability Statement

The datasets presented in this study can be found in online repositories. The names of the repository/repositories and accession number(s) can be found below: https://www.ncbi.nlm.nih.gov/genbank/, PRJNA575161.

## Author Contributions

WW carried out the experimental work, data analysis, and wrote the manuscript. JT carried out sample collecting and assisted in the laboratory work. KY, CH, JW, PL, HC, and BX helped in data analysis and the writing of the manuscript. QS and CZ supervised and contributed to the design of the experiments and revised the manuscript. All authors contributed to the article and approved the submitted version.

## Conflict of Interest

The authors declare that the research was conducted in the absence of any commercial or financial relationships that could be construed as a potential conflict of interest.

## Footnotes

1 https://www.arb-silva.de
