# Supplementary material for: Vertical Stratification of Dissolved Organic Matter Linked to Distinct Microbial Communities in Subtropic Estuarine Sediments
Source: Front Microbiol. 2021 Jul 20;12:697860. doi: 10.3389/fmicb.2021.697860 (PMC8329499; doi:10.3389/fmicb.2021.697860)
Supplement: Supplementary file 1 [file Data_Sheet_1.docx]

Supplementary Material for

**Vertical stratification of dissolved organic matter linked to distinct microbial communities in subtropic estuarine sediments**

**Wenxiu Wang^1^, Jianchang Tao^2^, Ke Yu^3^, Chen He^4^, Jianjun Wang^5^, Penghui Li^6,7^, Hongmei Chen^8^, Bu Xu^2^, Quan Shi^4,9*^, Chuanlun Zhang^2,9,10,11*^**

^1^State Key Laboratory of Marine Geology, Tongji University, Shanghai, China

^2^Department of Ocean Science & Engineering, Southern University of Science and Technology, Shenzhen, China

^3^School of Environment and Energy, Shenzhen Graduate School, Peking University, Shenzhen, China

^4^State Key Laboratory of Heavy Oil Processing, China University of Petroleum, Beijing, China

^5^State Key Laboratory of Lake Science and Environment, Nanjing Institute of Geography and Limnology, Chinese Academy of Sciences, Nanjing, China

^6^School of marine science, Sun Yat-sen University, Guangzhou, China

^7^Southern Marine Science and Engineering Guangdong Laboratory (Zhuhai), Zhuhai, 519082, China

^8^State Key Laboratory of Marine Environmental Science, College of Ocean and Earth Sciences, Xiamen University, Xiamen, China

^9^Shenzhen Key Laboratory of Marine Archaea Geo-Omics, Southern University of Science and Technology, Shenzhen, China

^10^Southern Marine Science and Engineering Guangdong Laboratory (Guangzhou), Guangzhou, China

^11^Shanghai Sheshan National Geophysical Observatory, Shanghai, China

***Corresponding Author**

*Quan Shi.* E-mail address: sq@cup.edu.cn

*Chuanlun Zhang.* E-mail address: zhangcl@sustech.edu.cn

**Supporting Figures**

**Figure S1.** DOM composition grouped by elemental (C, H, O, N, S) (a) and types of organic matter (b) based on the H/C and O/C ratios of DOM formulae according to Yuan, et al. (2017).

**Figure S2.** Distance-based redundancy analyses (dbRDA) of DOM composition with four factors selected for optimal dbRDA model by step analysis (a) and the influences of microbial and environmental drivers on DOM composition (b). ASV_PCo1 represents the first principal coordinate axis of microbial community.

**Figure S3.** Co-occurrence analysis of microbial amplicon sequence variants (ASVs) and DOM molecules with relative abundance over than 0.05% based on Pearson’s correlation analysis. Node sizes are proportional to the number of significant connections (Pearson’s correlation with *r* ≥ 0.65, *P* < 0.01) (degree). The color of edges indicates distinct targets to DOM compounds (Light brown: Aliphatic/proteins, Purple: Lignins/CRAM-like structures, Pink: Lipids and Green: Tannins). Followed are the calculated topological parameters in Gephi, average degree: 14.852, diameter: 6, density: 0.032, average length: 2.557.

**Figure S4.** Van Krevelen diagrams for DOM molecules (relative abundance ≥ 0.05%) significantly associated (Pearson’s correlation with *r* ≥ 0.65, *P* < 0.01) with bacterial (a) and archaeal (b) groups. #Correlation, the number of correlated amplicon sequence variants (ASVs).

**Figure S5.** Maximum likelihood phylogenetic tree of bacterial genomes based on 90 bacterial maker genes with abundance (Log_2_ (reads per million)) of genomes across six depths. Black: Reference genomes. Red: Genomes assembled from this study. Grey cicles: bootstrap values > 50%.

**Figure S6.** Maximum likelihood phylogenetic tree of archaeal genomes based on 105 archaeal maker genes with abundance (Log_2_ (reads per million)) of genomes across six depths. Black: Reference genomes. Red: Genomes assembled from this study. Grey cicles: bootstrap values > 50%.

**Figure S7.** Abundance of carbohydrate-active enzymes (CAZymes) (a and b) and ratios between auxiliary activity (AA) and glycosyl-hydrolases (GH) families among bacterial and archaeal genomes (c). **, *P* < 0.01; *, *P* < 0.05 (non-parametric Mann-Whitney test). In (a), the center line of each box indicates the median and whiskers indicate the smallest and largest values. The dots represent outliers.

**Figure S8.** Depth comparison in abundance of major carbohydrate-active enzymes (CAZymes) and corresponding microbial composition.

**Figure S9.** Abundance and microbial composition of enzymes or genes involved in lignin/aromatic degradation.

**Supporting Text S1**

**Phylogenetic analysis of bacterial and archaeal genomes**

To estimate the abundance of individual genomes across different depths, contigs from all binned genomes were mapped using Salmon (v.0.7.2; quasi-mapping) (Patro et al., 2017) by calculating reads per million and normalized per genome by the genome size. Phylogenetic affiliations were analyzed using the GTDB-Tk (v.0.1.6) genome-based taxonomy (Parks et al., 2018). Sequences closely related to our genomes were extracted from GTDB (v.89.0; http://gtdb.ecogenomic.org/) as the reference genomes. Phylogenetic tree was then built as described in Tully (2019). Briefly, reconstructed and reference genomes were provided as input to search 122 archaeal or 120 bacterial marker genes using HMM models downloaded from GTDB. All proteins identified as single copy maker genes were aligned using MUSCLE (v.3.8.31) (Edgar, 2004) with default setting and automatically trimmed using trimAL (v.1.4.rev15) (Capella-Gutierrez et al., 2009): -automated1. Single copy genes distributing in ≥ 30 genomes (n = 105 for archaea and 90 for bacteria) were concatenated and a phylogenomic tree was generated using FastTree (v.2.1.10) (Poon et al., 2010): -gamma -lg. The trees were visualized using the Interactive Tree of Life (iTOL, https://itol.embl.de) webtool.

**Text S2**

**Phylogenetic diversity in Pearl River estuarine sediments.**

Six samples from different depths (5 cm, 20 cm, 70cm, 130 cm, 200 cm, 300 cm) were selected for metagenomic sequencing. After assembly and tetranucleotide and coverage binning, 51 archaeal and 166 bacterial genomes with high completeness and low contamination (≥ 70% completeness and ≤ 5% contamination) were obtained (Table S7). Phylogenetic placement of the reconstructed genomes was determined using 90 bacterial maker genes and 105 archaeal maker genes. The 217 genomes were taxonomically diverse and comprised a total of 7 archaeal and 27 bacterial phylogenetic linages (Figure S5 and S6). Most of them were affiliated to archaeal linages of Bathyarchaeia (23), other Thermoplasmatota (10), MBG-D (5), Lokiarchaeia (4) and to bacterial linages of Desulfobacterota (25), Gammaproteobacteria (16), Planctomycetota (16) and Dehalococcoidia (14). Desulfobacterota were further divided into Desulfobacterales (9), Desulfatiglanales (8), Desulfobulbia (5) and Syntrophobacteria (3). Depth preferences of bacterial and archaeal lineages were observed with Gammaproteobacteria and Desulfobulbia exhibited high abundance in the upper layer while Planctomycetota, Dehalococcoidia and Bathyarchaeia were enrich in the deeper layer.

**References**

Capella-Gutierrez, S., Silla-Martinez, J.M., and Gabaldon, T. (2009). trimAl: a tool for automated alignment trimming in large-scale phylogenetic analyses. *Bioinformatics* 25(15), 1972-1973. doi: 10.1093/bioinformatics/btp348.

Edgar, R.C. (2004). MUSCLE: multiple sequence alignment with high accuracy and high throughput. *Nucleic Acids Res* 32(5), 1792-1797. doi: 10.1093/nar/gkh340.

Jain, C., Rodriguez, R.L., Phillippy, A.M., Konstantinidis, K.T., and Aluru, S. (2018). High throughput ANI analysis of 90K prokaryotic genomes reveals clear species boundaries. *Nat Commun* 9(1), 5114. doi: 10.1038/s41467-018-07641-9.

Parks, D.H., Chuvochina, M., Waite, D.W., Rinke, C., Skarshewski, A., Chaumeil, P.A., et al. (2018). A standardized bacterial taxonomy based on genome phylogeny substantially revises the tree of life. *Nat Biotechnol* 36(10), 996-1004. doi: 10.1038/nbt.4229.

Patro, R., Duggal, G., Love, M.I., Irizarry, R.A., and Kingsford, C. (2017). Salmon provides fast and bias-aware quantification of transcript expression. *Nature methods* 14(4), 417-419. doi: 10.1038/nmeth.4197.

Poon, A.F.Y., Price, M.N., Dehal, P.S., and Arkin, A.P. (2010). FastTree 2 – Approximately Maximum-Likelihood Trees for Large Alignments. *PLoS ONE* 5(3). doi: 10.1371/journal.pone.0009490.

Tully, B.J. (2019). Metabolic diversity within the globally abundant Marine Group II Euryarchaea offers insight into ecological patterns. *Nat Commun* 10(1), 271. doi: 10.1038/s41467-018-07840-4.
